# Distorted Grids as a Spatial Label and Metric

**DOI:** 10.1016/j.tics.2015.12.004

**Published:** 2016-03

**Authors:** Francis Carpenter, Caswell Barry

**Affiliations:** 1 Institute of Neurology, University College London, London, UK; 2 Department of Cell and Developmental Biology, University College London, London, UK

## Abstract

Grid cells have been proposed to encode both the self-location of an animal and the relative position of locations within an environment. We reassess the validity of these roles in light of recent evidence demonstrating grid patterns to be less temporally and spatially stable than previously thought.

Grid cells are neurones whose firing forms a regular triangular pattern that covers the environment of an animal (Figure 1A) [1]. This periodic firing means the modular grid-cell system encodes spatial information with remarkable efficiency (Box 1) and has fostered two widely held beliefs concerning their function. First, that grid-cell firing encodes the position of an animal within an environment, providing a ‘label’ for self-location 2, 3. Second, that grid cells act as a neural metric for space, encoding the spatial relationship between multiple locations 1, 2, 3, 4, 5. As a spatial metric, grid-cell-based networks are thought to be the basis of path integration (updating of self-location representations based on perceived motion) 1, 2, 3 and vector-based navigation (determining the angle and distance to a goal) 4, 5. These beliefs assume that grid-cell activity is spatially and temporally stable 2, 3, 4, 5. However, recent evidence shows that grid patterns evolve across time, and can be distorted and fragmented in space 6, 7, 8, 9, 10, 11, 12. We review evidence that grid patterns are less regular than was previously thought, and reassess their potential to function as a spatial label and metric.

## Grid Patterns: Evolving, Distorted, Fragmented

**Figure 1:**
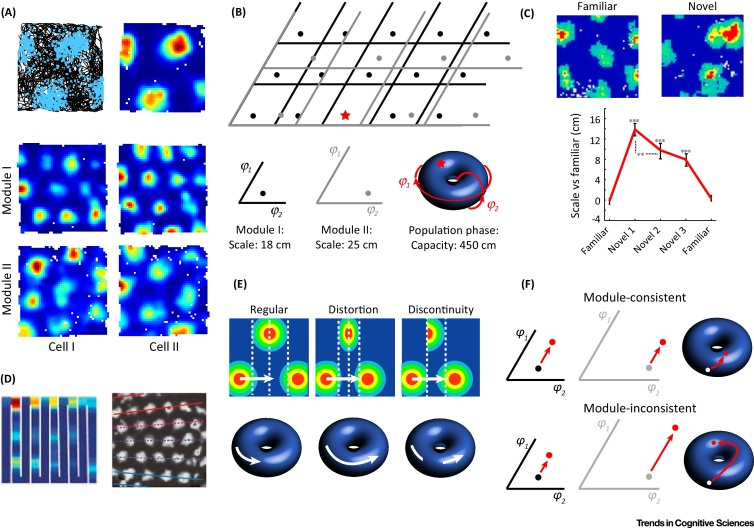
Grid Cell Variance in Time and Space, and its Implications. (A) Grid-cell recordings, showing raw data plots (top left): locations of action potentials in blue on the path of the animal, black. Firing-rate map for the same data (top right), hotter colours indicate higher firing rates, unvisited bins are white. Within a module, grid patterns share a common orientation and scale but have different firing locations (middle and bottom rows). Modules differ in their scale and possibly orientation (data adapted from [6] and [12]). (B) Distributed preferred firing locations within a module mean that in 2D self-location is encoded as a pair of phases: which cells are active within each module. Because grid-cell firing is periodic, the phase of each module repeats when the animal traverses a distance equal to its scale. The phase of each module therefore exists in a 2D phase-space with length equal to the scale of the module, and which wraps around at its edges (bottom). Because the phase of a single module repeats in environments larger than its scale, it provides only an ambiguous code for self-location (top). However, the conjunction of phases across two modules is here unambiguous, occurring only at a single position (the star). We term the conjunction of phases across modules the ‘population phase’. As a 2D periodic variable, population phase could also be plotted in a 2D space which wraps around at its edges. Alternatively, as here, the population phase can be represented continuously on the surface of a torus. (C) Grid patterns expand and become less regular when an animal first explores a novel environment (top), returning to a baseline configuration as the environment becomes familiar (bottom) (adapted from [6]). (D) In a hairpin maze, grid patterns fragment into discontinuous sub-patterns, which repeat across compartments (left, reprinted from [11] with permission from Nature Publishing Group). In a large environment, grid firing can be distorted, with firing being determined by local boundaries (right, reprinted from [8] with permission from Nature Publishing Group). (E) Effect of disruptions on the population phase. As an animal moves across the environment at a constant velocity (white arrow, top), the population phase changes at a constant rate (white arrow, bottom). Moving across a distortion in the grid pattern, the rate of change of the population phase per unit of distance moved increases or decreases. Moving across a discontinuity in the grid pattern, the population phase suddenly jumps between values. (F) If a disruption shifts the phase of each module (red arrow) away from its ‘true’ value in inverse proportion to the scale of the module, the population phase is shifted across the surface of the torus in proportion to the magnitude of the disruption. By contrast, if the disruption is inconsistent across modules, the population phase can jump to any point on the torus.

The triangular pattern and scale of each grid cell were originally reported to be consistent despite environmental change [1]. As such, the assumption that grid patterns are spatially and temporally invariant has underpinned subsequent theories of grid-cell function 2, 3, 4, 5. However, this supposition has been invalidated in two ways. First, grid firing evolves across time (Figure 1C): in novel enclosures grid patterns transiently increase in scale and become less regular, returning to baseline over several hours [6]. Second, environmental cues, particularly boundaries, can deform grid firing (Figure 1D). For instance, in strongly polarised enclosures such as trapezoids, grid patterns are locally rotated and rescaled [7]. Further, grid orientation can vary across larger enclosures, apparently influenced by local boundaries [8]. Indeed, if the boundaries of a familiar enclosure are moved, compressing or stretching the available space, grid patterns expand or contract concordantly 9, 10. Strikingly, the presence of boundaries which subdivide an enclosure can fragment grid firing into discontinuous patterns 11, 12. Importantly, protracted experience sometimes resolves such discontinuities [12]; it is unclear if other irregularities ameliorate over similar timeframes.

The extent to which disruptions in grid firing are consistent within and across modules remains undetermined. Given that cells within modules maintain their relative phases in different environments and after environmental deformations 9, 10, 13, we assume here that disruptions are consistent within modules. Conversely, concurrently recorded modules can maintain distinct orientations and respond independently to changes in environmental geometry 7, 8. This may reflect differences in the extent to which the firing of each module is determined by sensory and self-motion cues [14], and suggests that local disruptions in firing patterns may be inconsistent across modules.

## Grid Firing Robustly Labels Self-Location Despite Distortions

The number of unique population phases encoded by a grid-cell ensemble increases combinatorially with the number of grid modules (Box 1), meaning that grid cells provide an efficient label for self-location [2]. Hence a downstream network with access to each module's phase can accurately decode self-location provided that the population phase is sufficiently dissimilar at each location in the environment. Distortions and discontinuities, which respectively equate to shifts in the rate of change and sudden jumps in the population phase (Figure 1E), are immaterial unless they result in repetition of the population phase. Estimates of the capacity of the grid population of a rat are significantly larger than upper estimates of its ethological foraging area [2]. Thus, even if this area is encoded as a single map, distortions and discontinuities are likely only to generate different but still unique population phases across the environment.

Nevertheless, grid patterns do replicate when environments are compartmentalised 11, 12, likely reflecting the sensory equivalence of the compartments, a rare situation outside the laboratory. Without concurrent recordings, it is unknown if all modules replicate in the same way. If so, the population phase would also repeat, and grid firing alone would be insufficient to distinguish the compartments. However, grid patterns gradually distinguish perceptually-identical compartments, probably using self-motion [12], suggesting that potential ambiguities do not persist.

As a spatial label, specific population phases encode specific locations. Therefore, existing phase–location associations are invalidated when grid patterns evolve across time. To be robust to temporal changes in grid firing, a decoding network must be capable of updating the associations between population phase and location. Such a process is plausible, with similar dynamics presumably updating the associations from sensory cues to grid phase that maintain the short-term stability of evolving grid patterns, even while changes accumulate across hours or days 6, 12.

## Distorted Grids Introduce Metric Errors

Conceptions of grid firing as a spatial metric use the difference in population phase between two positions to calculate the vector connecting them in real-space 4, 5. This requires that all points separated by a consistent vector in real-space be separated by a consistent vector in phase-space. Thus, to be an accurate spatial metric, the population phase must change at a constant rate across the environment, as well as being unique at each location. Therefore, distortions and fragmentations in grid patterns (Figure 1E) will potentially introduce errors into navigation vectors that span those irregularities.

The nature of these errors depends primarily on whether disruption of the grid pattern is consistent across modules. If the distortion or discontinuity shift the phase of each module in inverse proportion to its scale, the encoded location will remain in agreement across modules. In such cases, the population phase will shift by an amount proportional to the size of the disruption in real-space (Figure 1F). Navigational vectors spanning such a region would be erroneous by an amount proportional to the magnitude of the disruption. By contrast, if the distortion or discontinuity affects modules inconsistently, the population phase can jump to any other value (Figure 1F). The resulting navigational errors would be disproportionate to the magnitude of the disruption and potentially catastrophic given the large capacity of the grid system.

Spare capacity in grid networks has been proposed to provide a form of error correction [15]. Such a scheme relies on the fact that, in enclosures smaller than the capacity of the grid population, a proportion of population phases are redundant, encoding locations outside the navigable enclosure. Disruptions resulting in invalid population phases could then in principle be identified as erroneous, with the phase returned to the most-recent or closest plausible value [15]. However, this requires a separate neural representation of which population phases are valid [15]. Thus errors stemming from misshapen grid patterns may only be corrected if they arise after the valid phases associated have previously been identified: distortions or discontinuities that appear on first exposure to an environment may be uncorrectable 7, 8.

In the theoretical framework considered thus far, the high capacity of the population phase means that the grid code is unambiguous in enclosures larger than the largest grid scale [2]. In an alternative framework, the largest module alone coarsely encodes self-location, although only unambiguously in enclosures smaller than its scale [3]. Smaller-scale modules are ‘nested’ within the largest module, providing increased resolution to the self-location code [3]. Because the phase in larger-scale modules resolves ambiguity in smaller-scale modules, the impact of disruptions in grid patterns again depends on how they are distributed across modules: distortions to a given module potentially render erroneous the contribution of all smaller-scale modules. Disruptions to small-scale modules would therefore result in small navigational errors, whereas disruptions to large-scale modules would potentially result in catastrophic errors.

In considering the implications of misshapen grids for metric decoding, we have assumed the decoding network to have limited capacity to account for such disruptions. While the possibility that all deformations are ‘mapped-out’ downstream of grid cells cannot be rejected, doing so would require accurate identification of the location, nature, and magnitude of all disruptions. If this were possible, it is unclear why the same information would not be used directly to correct grid firing. Indeed, that grid patterns regularise with experience suggests that disruptions can be resolved at the level of grid cells in particular conditions [12]. Conversely, if the decoding network could account for all disruptions, it is unclear why grid patterns would regularise.

## Concluding Remarks

Grid firing patterns evolve across time, and can be distorted and fragmented in space 6, 7, 8, 9, 10, 11, 12. Regardless, the capacity of grid population codes, together with the requirement only for a unique population phase at each location in the environment, make grid firing a robust label for self-location. By contrast, a requirement for a constant rate of change in population phase means using distorted or discontinuous grid patterns as a spatial metric is likely prone to errors. Although potentially introducing errors, disruptions to grid patterns do not exclude their being used as a metric. That grid patterns have been observed to exhibit experience-dependent regularisation [12] suggests that distortions and subsequent navigational errors may abate with protracted experience of an environment. To determine the full implications of misshapen grid patterns, future research should employ large-scale recordings to identify the number and relative scale of grid modules, whether they are disrupted independently of one another, the capacity for experience of an environment to ameliorate distortions, and how disruptions scale in environments of different sizes. In addition, concurrent testing of the types of errors made in spatial navigation tasks could provide behavioural evidence for the use of grid patterns as a spatial metric, and help to illuminate the mechanisms by which grid patterns are decoded.

## Box 1 Properties of Grid Cells in 2D, As Commonly Assumed by Models

Grid cells spike whenever the animal crosses the vertices of a triangular grid overlying the environment [1].

The firing pattern of each cell is defined by its scale (distance between firing peaks), orientation (angle of the grid), and phase (location of peaks) (Figure 1A).

Grid cells are organised into functional modules 9, 10, 13.

Within modules, cells share a common orientation and scale, but their firing locations are evenly distributed across space. A grid module therefore encodes self-location as a pair of phases in a 2D phase-space: which cells are active within each module.

Because grid firing is periodic, the phase of each module repeats after distances greater than its scale. Consequently, the phase of a module is ambiguous in environments larger than its scale.

However, because modules differ in scale, the conjunction of phases across different modules (the population phase) can resolve this ambiguity (Figure 1B).

The number of unique population phases increases with the number of modules. In principle, this means that grid cells can unambiguously encode self-location in enclosures much larger than the largest grid scale [2], or in spaces smaller than the largest scale but with high resolution [3], depending on the scale of modules relative to each other and the environment.
